# Autophagy and proteasomes in thymic epithelial cells: essential bulk protein degradation systems for immune homeostasis maintenance

**DOI:** 10.3389/fimmu.2024.1488020

**Published:** 2024-10-25

**Authors:** Noritaka Yamaguchi, Yuki Takakura, Taishin Akiyama

**Affiliations:** ^1^ Department of Molecular Cardiovascular Pharmacology, Graduate School of Pharmaceutical Sciences, Chiba University, Chiba, Japan; ^2^ Laboratory for Immune Homeostasis, RIKEN Center for Integrative Medical Sciences, Yokohama, Japan

**Keywords:** thymus, thymic epithelial cells, T cells, autophagy, proteasomes

## Abstract

The thymus is a central organ that controls T cell development. Thymic epithelial cells (TECs) create a unique microenvironment essential for the differentiation of major histocompatibility complex (MHC)-restricted and self-tolerant T cells. TECs present a complex of self-peptides and MHC molecules (self-pMHCs) to immature T cells and regulate their survival and differentiation based on their affinity for self-pMHCs. The processing of self-peptides in TECs depends on bulk protein degradation systems, specifically autophagy and proteasomes. Studies using autophagy- and proteasome-deficient mouse models have demonstrated that these degradation systems in TECs are indispensable for maintaining immune homeostasis. Although autophagy and proteasomes are ubiquitous in nearly all eukaryotic cells, TECs exhibit unique characteristics in their autophagy and proteasome functions. Autophagy in TECs is constitutively active and independent of stress responses, while TEC proteasomes contain specialized catalytic subunits. This review summarizes the distinctive characteristics of autophagy and proteasomes in TECs and their roles in immune system regulation.

## 1 Introduction

The thymus is the central organ responsible for producing immunocompetent T cells. Within the thymus, thymic epithelial cells (TECs) create a specialized microenvironment for the generation of T cells that express major histocompatibility complex (MHC)-restricted and self-tolerant T-cell receptors (TCRs) (1, 2). TECs are categorized into two major subtypes based on their localization: cortical thymic epithelial cells (cTECs) and medullary thymic epithelial cells (mTECs) (3–5). Bone marrow-derived T cell progenitors enter the thymus through blood vessels at the corticomedullary junction of the thymic lobules. The CD4–CD8– double-negative (DN) immature thymocytes then migrate to the outer cortex, where they interact with cTECs and differentiate into CD4+CD8+ double-positive (DP) T cells that express a diverse repertoire of TCRs. These DP cells undergo positive selection through interactions with cTECs displaying self-peptide–MHC complexes (self-pMHCs) (6–8). During this process, cTECs present self-pMHCs to DP cells and facilitate their survival and differentiation into CD4 or CD8 single-positive T cells (CD4 or CD8 T cells) that express TCRs with moderate affinity for self-pMHCs. MHC class I (MHC-I) and MHC class II (MHC-II) molecules present self-peptides to CD8 and CD4 T cells, respectively. Positively selected T cells migrate into the medulla and interact with mTECs. mTECs express a wide variety of peripheral tissue-specific antigens, in part through the function of the transcriptional regulator Aire (9), and also by replicating peripheral tissue-specific gene expression programs, a function carried out by the recently identified subset of mimetic TECs (10). T cells bearing a TCR with a high affinity for self-pMHC on mTECs undergo apoptosis during negative selection (7, 9). Alternatively, strong TCR signaling facilitates translocation of self-reactive CD8 T cells from thymus to the periphery and their development into mature and self-tolerant clones (11). Interactions between T cells and mTECs also promote the differentiation of immunosuppressive regulatory T cells (Tregs) in the thymus (12, 13).

The generation of self-peptides in TECs depends on bulk protein degradation systems, such as autophagy and proteasomes, which are ubiquitously present in eukaryotic cells and regulate various biological processes, including cell survival, proliferation, differentiation, and death (14). Compared with dendritic cells, which rely on the capture of exogenous proteins by endocytosis for processing of peptides for MHC-II, TECs are less active in endocytosis to present exogenous peptides (15, 16). Therefore, it is reasonable that TECs utilize cytoplasmic protein degradation systems, i.e. autophagy and proteasomes, for processing of self-peptides from endogenous self-antigens. Interestingly, autophagy and proteasomes in TECs exhibit unique characteristics compared with those in other cell types. While autophagy activation in most cells depends on cellular stress, autophagy in TECs is constitutively active and appears to be independent of stress responses (17, 18). Additionally, proteasomes in TECs contain unique catalytic subunits (19, 20). These distinctive characteristics are crucial for the processing of self-peptides and the selection of T cells (14). This review focuses on the molecular processes and functions of autophagy and proteasomes in TECs.

### 1.1 General Mechanisms of Autophagy

Autophagy, originally identified as a process to accelerate material and energy recycling, involves the digestion of proteins and organelles via autophagy-specific double-membrane vesicles that fuse with lysosomes, forming autophagosomes. This non-selective bulk autophagy is known as macroautophagy (21–23). Under non-starvation conditions, the mechanistic/mammalian target of rapamycin (mTOR) complex (mTORC1) suppresses autophagy by inactivating Unc51-like kinase 1 (ULK1), a protein kinase implicated in autophagy initiation. Starvation reduces mTOR activity, leading to the activation of ULK1 (24, 25). Activated ULK1 then phosphorylates proteins involved in autophagy initiation, including beclin-1, a key protein that activates vacuolar protein sorting 34 (VPS34) (26). VPS34, a catalytic subunit of class III phosphatidylinositol 3-kinase (PI3K), plays a role in endocytosis, intracellular vesicular trafficking, and autophagosome formation (27).

Autophagy is not limited to starvation; it is also activated by various forms of cellular stress, such as mitochondrial damage, oxidative stress, and hypoxia. The selective autophagy of mitochondria, known as mitophagy, has gained considerable attention (22, 28). Mitochondrial damage, primarily caused by reactive oxygen species (ROS), causes mitophagy via phosphatase and tensin homolog (PTEN)-induced putative kinase 1 (PINK1) and the E3 ubiquitin ligase, Parkin (29, 30).

### 1.2 Role of autophagy in TECs in T cell selection and development

Autophagy in TECs has been primarily observed in autophagy reporter transgenic mice that ubiquitously express a green fluorescent protein (GFP)-fused form of microtubule-associated protein light chain 3 (LC3), which is crucial for autophagosome formation. In TECs, constitutive activation of autophagy occurs even in the absence of starvation or infection, indicating that autophagy is triggered by stress-independent mechanisms (17). As TECs are less active in endocytosis to present exogenous peptides, autophagy is likely to highly active to process endogenously expressed self-peptides in TECs (15, 16). Studies of purified thymic stromal cells revealed that cTECs exhibited higher autophagic activity than mTECs. The role of autophagy in TECs was initially investigated using autophagy-deficient mice with a targeted disruption of *Atg5*, a gene essential for autophagosome formation (18). Transplantation of an *Atg5*-deficient thymus into athymic nude mice showed that autophagy deficiency did not affect overall T cell development. However, it impaired the shaping of the T cell repertoire and led to severe colitis and multi-organ inflammation in the recipient nude mice, indicating that autophagy in TECs is required for the selection of the T cell repertoire and the establishment of tolerance (18). While this study could not determine whether self-tolerance induction was ascribed to autophagy in mTECs or cTECs, subsequent studies suggested that autophagy supports the loading of antigens expressed by Aire+ mTECs onto MHC-II for the negative selection of CD4 T cells (31).

The role of autophagy in MHC class II-mediated CD4 T cell development has also been explored in mice lacking genes crucial for lysosomal-autophagosomal proteolysis and autophagosome formation. The lysosomal endopeptidase cathepsin L is highly expressed in cTECs and regulates the processing of MHC-II invariant chain (Ii) and generation of self-peptides for MHC-II-mediated CD4 T cell development (32, 33). Endosomal and lysosomal thymus-specific serine protease (TSSP) contributes to the diversification of the functional CD4 T cell repertoire in the thymus (34, 35). Lysosomal-associated membrane protein 2 (LAMP2) is a lysosomal protein that promotes the fusion of autophagic vacuoles with lysosomes during macroautophagy (36, 37). LAMP2 is highly expressed in cTECs. Although deletion of *Lamp2* in thymic stromal cells did not affect the overall differentiation of cTECs and mTECs, it specifically impaired CD4 T cell development without affecting the CD8 lineage. Mechanistically, inactivation of autophagy in *Lamp2*-deficient cTECs caused defects in MHC-II processing, leading to a marked reduction in CD4 TCR repertoire diversity (38).

**Figure 1:**
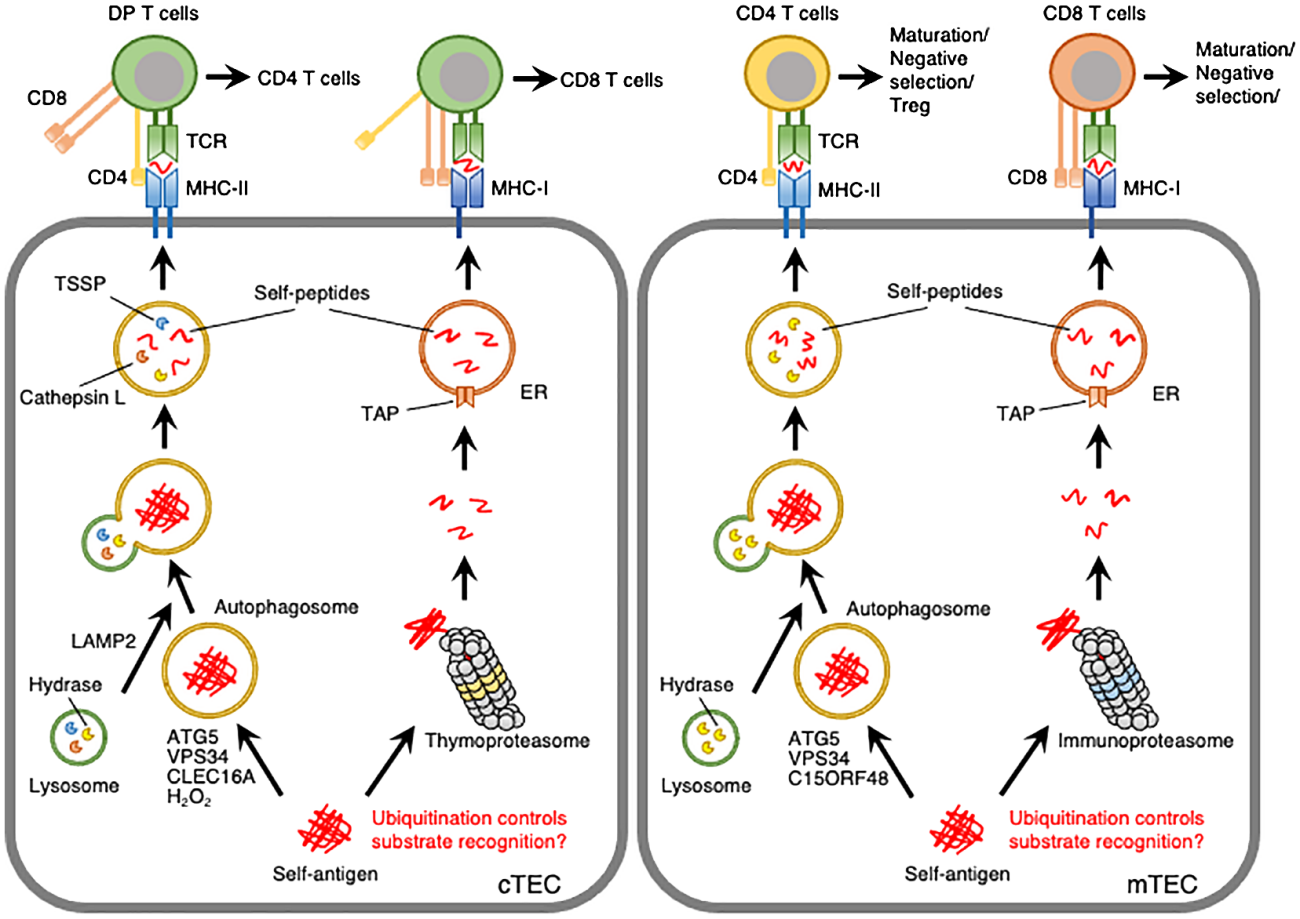
Model depicting autophagy- or proteasome-mediated processing of self-peptides in cTECs and mTECs. Self-antigens expressed in TECs are processed into self-peptides by autophagy or proteasomes and then loaded onto MHC-II or MHC-I molecules, respectively. ATG5 and VPS34 are required for autophagy in cTECs and mTECs. LAMP2, CLEC16A, and hydrogen peroxide (H_2_O_2_) are involved in autophagy in cTECs. C15ORF48 is closely associated with autophagy initiation in mTECs and, to a lesser extent, in cTECs. Thymoproteasomes and immunoproteasomes are expressed in cTECs and mTECs, respectively, with distinct catalytic subunits and substrate-processing activities. Post-translational modifications, such as ubiquitination, may be involved in substrate recognition by autophagy and proteasomes.

The role of VPS34, a catalytic subunit of class III PI3K, in TECs was elucidated by the TEC-specific disruption of *Vps34* in mice. Loss of VPS34 in TECs resulted in almost complete inactivation of autophagy in both cTECs and mTECs and abolished the positive selection of CD4 T cells without affecting CD8 T cells. TCR sequencing revealed that deletion of *Vps34* in TECs altered T cell repertoire properties and reduced clonal sharing. cTECs lacking *Vps34* exhibited an increased abundance of invariant chain intermediates bound to surface MHC class II molecules, indicating altered antigen processing (39). Collectively, autophagy deficiency due to the deletion of *Lamp2* or *Vps34* in TECs demonstrates that autophagy is indispensable for the processing of self-pMHC class II complexes in TECs and the generation of a broad CD4 TCR repertoire (**Figure 1**).

### 1.3 Molecular mechanisms of autophagy in TECs

While the importance of autophagy in TECs for T cell selection and development has been established in autophagy-deficient mouse models, the molecular mechanisms underlying autophagy in TECs remain unclear. Autophagy in TECs is constitutively active and should be dependent on specific mechanisms that may differ from those of typical autophagy (17, 18).

C-type lectin domain family 16A (CLEC16A) is a membrane-associated endosomal protein that functions as an E3 ubiquitin ligase and is implicated in various autoimmune disorders, such as multiple sclerosis, type 1 diabetes, and systemic lupus erythematosus (40). Genetic evidence has demonstrated the involvement of CLEC16A in autophagy in various cell types (41–43). A previous study showed that whole-body knockdown of *Clec16a* in non-obese diabetic (NOD) mice with type 1 diabetes reduced autophagic activity in cTECs and affected T cell selection in the thymus. Interestingly, while *Atg5* disruption causes autoimmunity, *Clec16a* knockdown mice exhibit T cell hyporeactivity and suppression of autoimmune phenotypes, even though both impair autophagy activity in TECs (44). Although the exact mechanism behind the suppression of autoimmunity by *Clec16a* deficiency is unclear, CLEC16A-dependent autophagy in TECs may regulate T cell maturation without significantly impacting the T cell repertoire.

mTOR is a key regulator of various biological processes, including cell growth, differentiation, and autophagy (45). The role of mTOR in TECs was investigated using pharmacological inhibition of mTOR kinase activity and TEC-specific deletion of *Mtor*. The mTOR inhibitor rapamycin induces severe thymic atrophy and reduces the number of TECs. TEC-specific deletion of *Mtor* caused a significant reduction in mTECs, defects in thymocyte differentiation, and autoimmune phenotypes, such as multi-organ immune cell infiltration and autoreactive antibodies in sera. Mechanistically, deletion of *Mtor* in TECs led to upregulation of autophagy-mediated degradation of β-catenin and inactivation of Wnt/β-catenin signaling, suggesting that mTOR promotes TEC development and maturation by suppressing autophagy-mediated over-degradation of β-catenin (46). Since the role of mTOR in autophagy-dependent processing of self-peptides in TECs remains unclear, further studies on the role of mTOR in TECs are needed.

H_2_O_2_ is a relatively long-lived, cell-permeable ROS that acts as an inducer of autophagy (47). H_2_O_2_ activates AMP-activated protein kinase (AMPK) in conjunction with a decrease in the ATP: AMP ratio (48), leading to ULK1-mediated autophagy. Additionally, H_2_O_2_ enhances the oxidation of ATG4, thereby inactivating the LC3-delipidation activity and promoting autophagosome formation (49). H_2_O_2_ also initiates autophagy by altering the thiol redox state (50), inhibiting mTOR signaling (51), and inducing beclin-1 expression (52). cTECs exhibit low expression of the H_2_O_2_-quenching enzyme catalase, suggesting the involvement of H_2_O_2_ in autophagy within these cells (53). This involvement has been studied using transgenic mice stably expressing mitochondria-targeted human catalase (mCat Tg). mCat Tg mice showed a significant reduction in mitochondrial H_2_O_2_ levels and autophagy in cTECs without affecting mTECs. Furthermore, these mice exhibited defects in the clonal deletion of thymocytes and developed autoimmune phenotypes, such as increased tissue infiltration of lymphocytic cells and serum antinuclear antigen reactivity (54). These findings suggest that the production of H_2_O_2_, facilitated by the low expression of catalase, induces constitutive autophagy and regulates the clonal deletion of T cells in cTECs (**Figure 1**).

Recently, our studies have demonstrated that the mitochondrial protein chromosome 15 open reading frame 48 (C15ORF48) plays a crucial role in initiating autophagy in TECs (55). C15ORF48 functions as an accessory subunit of the electron transport chain complex IV, where it suppresses cytochrome *c* oxidase activity (56, 57). We identified C15ORF48 as an inducer of autophagy in human lung cancer cells. Notably, C15ORF48 promotes autophagy independently of mTOR phosphorylation, which is an indicator of starvation stress, or mitophagic activity. This suggests that C15ORF48 initiates autophagy independently of starvation stress or mitochondrial damage. C15ORF48 has been shown to lower the mitochondrial membrane potential and reduce intracellular ATP levels, thereby activating the pro-autophagic signaling pathway AMPK-ULK1. Single-cell RNA sequencing analysis of thymic cells revealed high expression levels of *C15orf48* in both cTECs and mature mTECs. Importantly, whole-body *C15orf48* knockout mice showed significant reduction in autophagy in mTECs and, to a lesser extent, in cTECs. Moreover, *C15orf48*-deficient mice exhibited autoimmune features, including the production of self-reactive antibodies in the serum, multi-organ infiltration of inflammatory cells, and glomerulitis-like IgG accumulation in the kidneys. Transplantation of *C15orf48*-deficient thymic stroma into athymic nude mice resulted in similar autoimmune phenotypes. Global T cell development remained largely unaffected by *C15orf48*-deficiency, except for a slight reduction in mature CD4 T cells. Thus, this study highlights the role of C15ORF48 in the induction of stress-independent autophagy in TECs, likely independent of mitophagy, and suggests it may shape the T cell repertoire by regulating T cell selection, particularly in mTECs (55) (**Figure 1**). Future studies are needed to explore the impact of *C15orf48*-deficiency on the T cell repertoire.

### 1.4 Thymoproteasomes in cTECs

Proteasomes are multicatalytic complexes that regulate protein degradation. In general, ubiquitination of target proteins is a hallmark of proteasome-mediated degradation (58). The cytosolic proteasome is a 26S protein complex consisting of a 20S enzymatic core particle flanked by two 19S regulatory particles. The 19S regulatory particles recognize ubiquitinated target proteins. The 20S core particle is a multicatalytic protease complex composed of 28 subunits arranged cylindrically into four heteroheptameric rings: α1-7, β1-7, β1-7, and α1-7. Within the proteasome, the β1, β2, and β5 subunits function as catalytic components (58). Peptides processed by proteasomes in the cytosol are translocated into the lumen of the endoplasmic reticulum (ER) by a transporter associated with antigen processing (TAP) and subsequently loaded onto MHC-I molecules (59). cTECs uniquely express the catalytic subunit β5t (*Psmb11*), which forms a specialized proteasome known as the thymoproteasome (19). The β5t subunit has altered proteolytic activity that leads to the preferential cleavage of proteins at hydrophilic peptide residues, reduced chymotrypsin-like activity, and reduced enzyme kinetics compared with β5 (60). The β5t-containing proteasome generates peptides with not only different amino acid residues but also different quantities from those produced by other proteasomes, and these quantitative and qualitative differences may lead to the presentation of unique MHC-I-associated peptides (61, 62). Targeted disruption of *Psmb11* leads to defects in the maturation of CD8 T cells, alterations in the TCR repertoire, and impaired T cell responses (19, 63, 64). Since proteasomes in TECs are crucial for generating peptides presented by MHC-I molecules (65), these findings indicate that thymoproteasomes contribute to the generation of self-peptides necessary for the positive selection of CD8 T cells (**Figure 1**).

### 1.5 Immunoproteasomes in mTECs

mTECs express a different type of proteasome, the immunoproteasome, which contains the catalytic subunits β1i (*Psmb9*), β2i (*Psmb10*), and β5i (*Psmb8*) (20, 62). *Psmb8, Psmb9, and Psmb10* triple knockout mice showed a reduction in CD8 T cells and decreased expression of MHC-I in the thymus. Mass spectrometry analysis of peptides associated with MHC-I revealed that immunoproteasome deficiency altered the MHC-I-associated peptide repertoire (66). The peptide-processing-independent role of the immunoproteasome in mTECs was shown in *Psmb8* and *Psmb10* double knockout (dKO) mice. These mice exhibited low expression of *Psmb9*, which rendered them almost complete deficiencies in immunoproteasomes. dKO mice exhibited accelerated thymic involution, characterized by a reduction in CD8 T cells and mTECs, as well as the induction of multiorgan autoimmune manifestations. Loss of *Psmb8* and *Psmb10* led to proteotoxic stress in mTECs, resulting in the exhaustion of postnatal mTEC progenitors (67). These findings suggest that the immunoproteasome plays a crucial role in generation of MHC-I-associated self-peptides and maintaining mTEC homeostasis, thereby supporting MHC-I-mediated CD8 T cell selection.

The thymus lacking thymoproteasomes and immunoproteasomes was examined in *Psmb8, Psmb9, Psmb10, and Psmb11* quadruple knockout (4KO) mice. The 4KO mice showed a severe defect in generation of CD8 T cells without affecting that of CD4 T cells. Loss of thymoproteasomes and immunoproteasomes increased apoptosis-dependent negative selection of CD8 T cells in the thymus (68). These results suggest that the restricted expression of thymoproteasomes and immunoproteasomes in the thymus is likely to promote a switch in the self-peptides presented by cTECs and mTECs. The difference in self-peptides displayed in the cortex and the medulla may be crucial for eviction of positively selected thymocytes from subsequent negative selection and the development of CD8 T cells (62).

## 2 Discussion

Autophagy- or proteasome-deficient mouse models have demonstrated the indispensability of these proteolytic systems in TECs for the processing of self-peptides and the selection and differentiation of T cells. However, molecular details about how autophagy and proteasomes in TECs selectively degrade self-peptides have been largely unknown. Most likely, the reason of this limitation is the difficulty in isolation of enough amount of primary TECs from thymus for biochemical and cell biological analyses (16, 69). Ubiquitination functions as a signal for substrate recognition in autophagy as well as proteasome-mediated degradation (22, 58). Ubiquitinated proteins interact with the autophagy machinery through autophagy adaptors and promote autophagosome formation (70). Therefore, it is plausible that the ubiquitination of self-peptides regulates their processing by autophagy and proteasomes in TECs. However, whether self-peptides are ubiquitinated in TECs remains unclear. As CLEC16A can associate with ubiquitin ligases and deubiquitinases (71–73), CLEC16A may be involved in ubiquitination of self-peptides in TECs. A study on the post-translational modifications of self-peptides in TECs would be instrumental in understanding the mechanisms underlying autophagy-mediated selective processing of self-peptides.

Recent studies have revealed that mTECs express lineage-defining transcription factors and convert themselves into mimetic TECs that possess characteristics of various peripheral-tissue cells (10). It is obscure whether these mimetic TECs utilize constitutive autophagy and immunoproteasomes for processing of self-peptides as observed in mTECs. Given that Aire is involved in accumulation of mimetic TECs (10) and that *C15orf48* is highly expressed in Aire+ mature mTECs (55), mimetic TECs may have at least C15ORF48-dependent constitutive autophagy for processing of self-peptides. This hypothesis should be examined by isolating mimetic cells from thymus.

In conclusion, considering the importance of autophagy and proteasomes in TECs for the development and selection of immunocompetent T cells, understanding the molecular mechanisms of these protein degradation systems is essential for gaining insights into the nature of the immune system and immune-associated disorders. Therefore, the relationship between autophagy and proteasomes in TECs and human autoimmune diseases warrants further investigation.
